# A dynamic nonlinear flow algorithm to model patient flow

**DOI:** 10.1038/s41598-025-96536-z

**Published:** 2025-04-08

**Authors:** Arsineh Boodaghian Asl, Jayanth Raghothama, Adam S. Darwich, Sebastiaan Meijer

**Affiliations:** Department of Biomedical Engineering and Health Systems, KTH Royal Institute of Technology, Huddinge, 14157 Sweden

**Keywords:** Health care, Health policy

## Abstract

Hospitals are complex systems, and the flow of patients is dynamic and nonlinear in such systems. Network representation allows flow algorithms to observe bottlenecks as candidates for optimisation. To model the dynamic behaviour of the patient flow, we need to consider the variability in arrival rates and service times (length of stay). Previously proposed dynamic flow algorithms mainly focused on arrival and departure rates, inflow and outflow, edges’ and vertices’ capacity, and routing, with applications mainly in transportation and telecommunication. In hospitals, bottlenecks that emerge from the patients’ flow are a result of the vertices (wards) behaviour defined by capacity (beds), number of servers (staff), service time variability, and edges (care pathways) distribution probability. We offer a modified flow algorithm that takes a hospital network, iterates over the patients’ arrival rates, and measures the flow with respect to vertices’ capacities, servers, service time variability, edge capacity, and distribution probability. The result is a dynamic residual graph to measure the bottlenecks’ persistency and severity, identify the root causes of bottlenecks, and wards’ dynamic nonlinear behaviour. The algorithm provides a quick holistic view of hospital performance and the analysis of the edges and vertices’ behaviour over time.

Patients’ flow behaviour in a hospital is dynamic and nonlinear due to the uncertainty and variability in arrival rates and the service time duration^1–6^. The arrival rate variability depends on external factors such as emergencies, unforeseen situations, or seasonal changes. In contrast, service-time variability depends on internal factors such as the number of staff in each ward or patients’ behaviour^1–4,6,7^. Patients’ care pathways and length of stay in a hospital depending on their health issues and service time variability^1,4,5,8^. The arrival of irregular patients from multiple pathways and the uncertainty in their service time cause nonlinear behaviour on each ward^9^. Therefore, the bottleneck in a ward can have roots in other wards^8,10^. A holistic view of a hospital is required to capture the dynamic behaviour and the source of bottlenecks^11^.

Flow algorithms are well suited to analyse complex systems and identify bottlenecks based on residual capacity^12,13^. The residual capacity is defined by reducing the utilised capacity from the total capacity. The purpose of the flow algorithm is to measure the flow through path searching and augmentation to identify the maximum flow and residual capacities for decision-making and optimisation. It handles complex system modelling through the initiation and termination of the flow, path augmentation, and capacity adjustment^14,15^. However, arrival rate variability, number of servers, service time variability, edges and vertices’ capacities, and distribution probability are necessary to model the flow of the patients using the algorithm^1,2^.

Hospitals can be viewed as a network of vertices (wards) connected through edges (care pathways) where each vertex has a capacity (beds) and servers (staff), and each edge has a capacity and distribution probability. When patients arrive at the hospital, they follow some edges and vertices based on their health issues. The pattern in which patients enter the hospital indicates the arrival rate and varies over time^16^, and the patients’ diffusion throughout the hospital defines the edge distribution probability. These two factors help to measure the edge inflow rate. However, each patient spends an uncertain amount of time in wards, which depends on the individual’s service time and the number of servers. Servers distribute the workload in each ward and reduce the average service time^9^ nonlinearly. These two factors define the service rate and affect the flow intensity based on the arrival rate, which indicates whether a ward can handle more patients. For a patient to stay in a ward, there should be available capacity. When a ward is full, the patients either wait in a queue or remain in the previous ward^17^. Hence, the time for a patient to arrive and leave the hospital can vary significantly depending on these factors^2,18,19^. Modelling the patients’ flow through all the edges and vertices over time can provide a holistic view of these factors’ interrelations and their variability^7,8^ to observe the nonlinear dynamic behaviour and bottlenecks’ persistency and severity.

The network representation of a hospital allows flow algorithms to analyse patients’ flow and perceive the overflow state for decision-making. The first static^20^ and dynamic flow^21^ algorithms were proposed by Ford and Fulkerson to solve the transportation problem by finding the maximum flow through path augmentation. The primary purpose was to enhance transportation by determining the paths with the least congestion. The dynamic flow algorithm aims to observe the dynamic behaviour of a system over time by creating a continuous flow^21^. Flow algorithms primarily measure the maximum amount of the flow, which initiates from a given source vertex and terminates in a given target vertex through path augmentation and sending flow through residual capacity^12^. Many proposed dynamic flow algorithms aim to observe the flow behaviour mainly based on some parameters’ variability, such as arrival and departure rates^18,22,23^, vertex and edge capacities and variability^24–26^, inflow and outflow rates^23,25^. They allow the investigation of the flow behaviour over time for transportation, telecommunication, and evacuations^19,20,23,27^, where the edge capacity still plays a significant role. Contrary to this, patients’ flow behaviour in a hospital is not only based on the variability of the arrival rate but is significantly influenced by the wards’ capacity, number of servers, and service time variability, where an edge capacity is for patients to wait, therefore, allocating them indicates the ward is at an overflow state. However, it is the distribution probability of each edge that affects the inflow rate and intensity. In general, routing in a flow algorithm is either due to congestion or desire^15,28,29^. However, in a hospital system, patients arrive continuously and unexpectedly^4–6^ when wards are mostly occupied. The determination to transfer patients is based on individual health issues and not the shortest path to the target ward. Patients may be required to visit all the necessary wards to complete their health procedure, irrespective of the bottlenecks. This requires the proper modification and adjustment of network algorithms when modelling and analysing complex health systems to increase the validity of the output behaviour^30,31^. Therefore, we modified the flow algorithm to consider the effect of these parameters to measure the continuous. Hence, for the algorithm to model the patient flow properly, we use a network representation of a hospital and adjust both the vertices and edges capacities based on the provided arrival rate, distribution probability, and service times, which vary for each patient, ward and time, then, measure the forward flow to identify the wards behaviour.

From the transportation perspective, vertices have been the road conjunction where vehicles choose an edge, and edge capacity has been the crucial parameter to focus on measuring congestion. Bottlenecks are caused by the traffic on the edge where vehicles are stuck either due to traffic light scheduling or delays in transportation arrival and departure time. A hospital system functions differently than a transportation system. Vertices in a hospital have a crucial role in delivering services to patients. Arrival and departure rates cannot be fixed parameters, and vertex capacities are not the only parameters to identify the bottlenecks. A service time is based on the number of available beds, the number of staff, and patient behaviour toward providing better healthcare. Therefore, both arrival rate variability and service time variability define the wards’ dynamic nonlinear behaviour. To optimise transportation, adding, removing, and reversing edges are the primary focus, whereas adding or removing a new ward and managing ward resources are the primary concerns of hospital management. Furthermore, routing in networks such as transportation is governed by either congestion or desire^15,28,29^; In a hospital, patients follow a pathway based on their health issue, likely defined at the arrival point, where each ward has a distinct functionality and purpose to accomplish, which requires the careful consideration of which path searching algorithm is proper to model patients’ flow in the hospital. Hence, to develop a dynamic nonlinear flow algorithm to model the patients’ flow, we consider the arrival rate variability and service time variability, with fixed vertex capacity, vertex servers, edge capacity, and distribution probability to capture the bottlenecks and nonlinear behaviour, where the proposed algorithm searches for longer paths to push flows through.

To model a continuous flow in any system using a dynamic flow algorithm can be challenging^25^. Using a flow algorithm in its default form can underestimate the system’s proper behaviour and the validity of the output^32^. This is because each system requires parameters where some are fixed, and some vary over time. Time or arrival rate variability has been the key parameter used to model a continuous flow. Researchers have investigated and proposed dynamic flow algorithms by considering parameters such as earliest arrival, latest departure, inflow and outflow rates, edge capacity variability, and time and arrival rate variability on both single-commodity and multi-commodity network^15,18,22,23,25,33^. Applying vertices’ capacities has been proposed by various researchers to model flows in systems by enhancing the maximal flow algorithm with time delay and variability at the edge^34,35^. It has also been shown that vertex capacity is not vital to consider for minimum cost flow algorithm^36^ but from a transportation perspective. Apart from parameters’ variability, few researchers have also considered the dynamic reversal of edge directions for evacuation^23^, dynamic removal and adding of edges to the road network^24,26^, and routing decision^28,29^ for transportation optimisation.

To this date, no published flow algorithm has addressed the modelling of patients’ flow. Furthermore, parameters such as number of servers, service time variability, and distribution probability have not been taken into consideration by previous dynamic flow algorithms. We offer a dynamic nonlinear flow algorithm that iterates over arrival rates to measure the flow. The algorithm generates a unique service time per vertex at each arrival rate for the individual patient, calculates the service rate by considering the number of servers based on the individual’s service time, and the flow intensity based on the arrival rate effect on service rate to adjust each vertex’s capacity, and calculates the effect of each arrival rate and distribution probability on edges inflow rate to adjust each edge’s capacity. The adjusted capacity is the sum of the adjusted vertex and edge capacities. The algorithm uses a depth-first search for path searching and augmentation and eliminates the backward flow to increase the validity of the uncovered bottlenecks. As a result, the algorithm provides a holistic view of the hospital bottlenecks’ persistency, severity, and overflow and the wards’ dynamic nonlinear behaviours.

## Methods

### Implementation

The first flow algorithm was proposed by Ford and Fulkerson^20^, where Edmonds and Karp introduce the path searching and augmentation to this algorithm using breadth-first search^37^. Our proposed algorithm is based on the Ford and Fulkerson algorithm but uses Edmonds and Karp’s path searching and augmentation with breadth-first search. Algorithm 1 represents the dynamic nonlinear flow pseudocode. The algorithm accepts a list of input parameters to construct the hospital network and assigns parameters to each edge vertex and edge. The purpose of the algorithm is to provide a dynamic residual graph to observe the bottlenecks’ persistency, severity, overflows, and dynamic behaviour based on the residual capacity. Following are the required input and output parameters and their selection purposes:

- **Directed Graph **(*DG*(*V*, *E*)): This represents the hospital network where the vertices $\{V_i\}_{i=1}^n \subseteq V$ are the wards, and the edges $\{E_i\}_{i=1}^m \subseteq \{(u, v) : u, v \in V\}$ are the care pathways for patients to flow from one ward to another. The graph allows us to define the wards’ connectivity and patients’ flow direction and embed parameters on edges and vertices. *n* is the maximum number of vertices, and *m* is the maximum number of edges. The **Source **($v_0$): This vertex indicates where the flow should originate, which is where patients enter the hospital. The **Sink** ($v_{n-1}$): This vertex indicates where the flow should terminate, which is where the patients leave the hospital. Since the algorithm is based on single-commodity, we have defined only one vertex for the flow source and only one vertex for the flow sink.
- **Arrival rates **($\{R\}_{i=0}^{t}$):. This indicates the frequency that patients arrive at the hospital per time unit, starting from time 0 to time *t*. The parameter allows us to justify the flow rate to patients’ arrival rate on edges and vertices.
- **Vertices’ capacities **($\{C_i\}_{i=1}^n$): This indicates the number of beds per ward. The vertices’ capacities are crucial for measuring the flow intensity and understanding the bottlenecks based on bed limitations.
- **Vertices’ distribution functions **($\{DF_i\}_{i=1}^n$): Generates a service time per patient for each ward per arrival rate within the given minimum and maximum length of stay $\{L_{v_i}^k\}_{i=1}^n$, *k* are the number of service times per vertex. Each ward has a unique distribution function assigned based on multiple length of stay variations. **Vertices’ servers **($\{S_i\}_{i=1}^n$): This indicates the number of staff per ward, which affects the service rate in each vertex and further the flow intensity.
- **Edges’ capacities **($\{Q_i\}_{i=1}^m$): This indicates the number of patients that wait in a queue upon the availability of a bed in an upcoming ward.
- **Edges’ distribution probabilities **($\{DP_i\}_{i=1}^m$): This indicates the likelihood that a patient may follow one of the outgoing edges, where the sum of the distribution probabilities of outgoing edges for each vertex is equal to 1. The lower the distribution probability, the less likely it is for those edges to cause bottlenecks.
- **Dynamic residual graph **(*DynRG*): This stores the minimum and maximum residual capacities for the given time interval to observe the bottlenecks’ severity, persistency, and overflows. **Dynamic vertex behaviour **(*DynV*): This stores the current capacities per vertex per time unit to observe the wards’ dynamic nonlinear behaviours.

The algorithm 1 first constructs a directed graph *DG* for the hospital network with the given vertices *V*, edges *E*, and aforementioned input parameters (lines 1 to 4). Next, we define a static *staRG* and a dynamic *DynRG* graphs to store the residual capacity (lines 6 and 7) and let the minimum residual capacity *min_cap* and maximum residual capacity *max_cap* to be infinity $\pm \infty$ respectively (lines 8 to 10). Next, in line 12, the algorithm iterates over the arrival rates *R*. We initialised the *StaRG* and *DynRG* graphs outside the arrival rates loop to model the patients’ flow continuously. Within this loop, we need to adjust the capacity using the input parameters. Hence, we construct a loop to iterate over each edge’s *E* vertices ${u} \rightarrow {v}$ to adjust the capacities based on the service rate and flow intensity (lines 14 to 24).

The purpose of capacity adjustment is to observe the flow state based on the input parameter, which is significantly affected by arrival rate and service time variation. To model the service time variation, we generate service times *sTime* per patient for each vertex based on their given distribution function *DF* each time the arrival rate changes *R* (lines 15 to 17). Each vertex has a unique distribution function *DF* defined based on its multiple lengths of stays. This allows us to assign a proper service time *sTime* for each ward within the range of the minimum and maximum length of stay *L*. The distribution function generates a random value sample for *sTime*, then the algorithm checks until it ensures the generated service time lies within the proper range of length of stay $[min(L_{v}), max(L_{v})]$ for the given vertex. The distribution function allows maintaining the service time variability per arrival rate to observe the dynamic nonlinear behaviour of patients’ flow.

**Algorithm 1:**
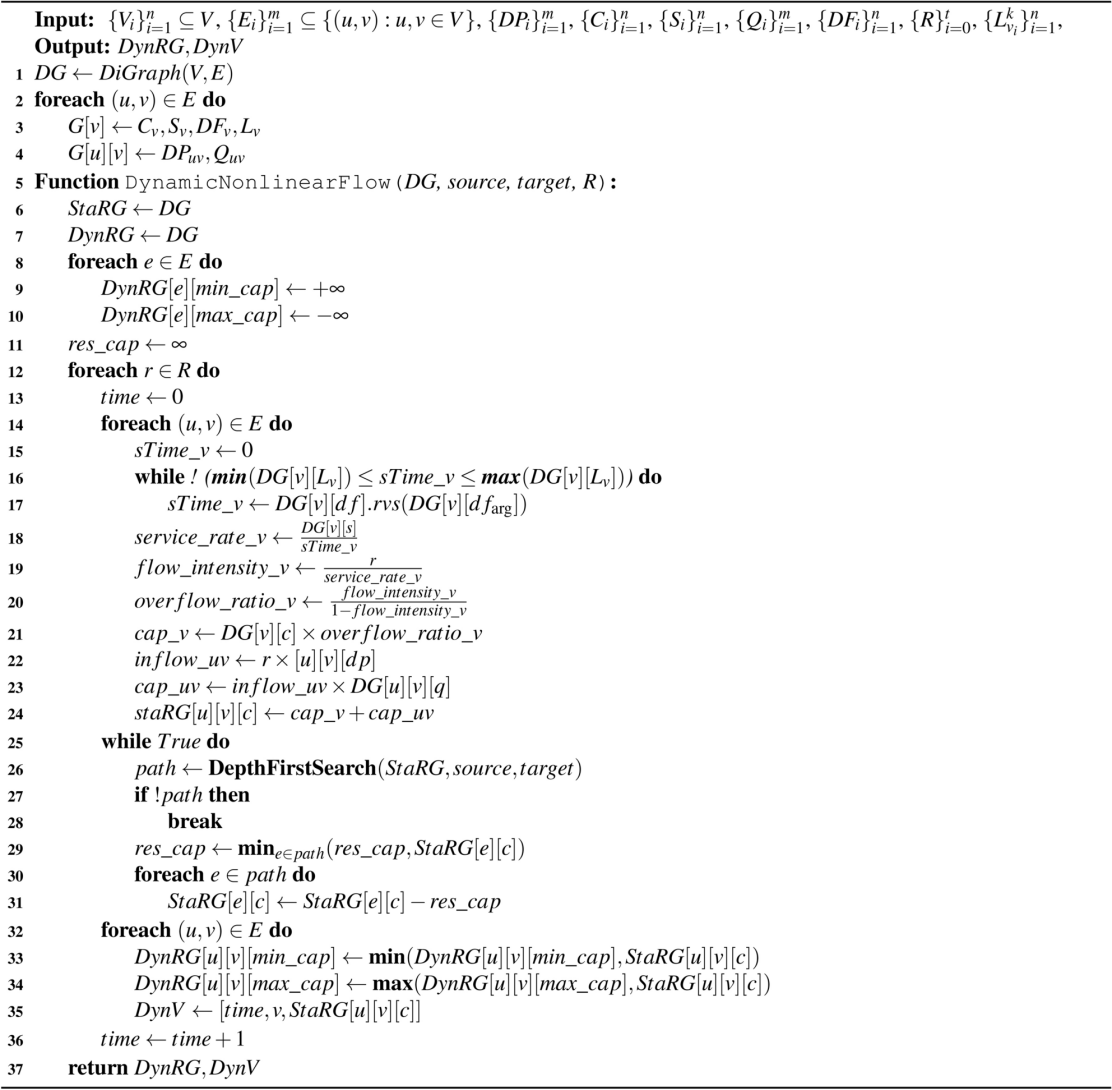
Dynamic Nonlinear Flow Algorithm.

To adjust the capacities, first, we calculate the service rate for each ward based on the number of servers *S* over the service time *sTime* per patient (line 18). This helps to measure the efficiency of each ward irrespective of the arrival rate; this is because the speed at which patients may leave a ward is based on staff availability and individual patients’ length of stay. However, the frequency at which patients arrive at each ward affects the wards’ performances. Through flow intensity, we can observe the effect of the arrival rate at each time $r_i$ on the service rate (line 19), which helps to measure the overflow state. For further clarification, a flow intensity of equal to 0 indicates there is no flow into the ward, a flow intensity between [0,1] indicates the ward is occupied but not overflowed, a flow intensity equal to 1 indicates the ward is at full capacity, and finally, a flow intensity of over 1 indicates the ward is overflowed. Hence, by subtracting flow intensity from 1, we can measure the intensity of the overflow. However, since there is no threshold on how large the flow intensity can grow positively, we measure the overflow state based on the ratio of the current flow intensity over the remaining flow intensity (line 20). Therefore, to perceive the utilisation of each ward, we adjust its capacity by measuring the effect of the overflow_ratio on each vertex’s capacity (line 21). Therefore, with a positive value, there will be capacity for the flow to pass, which enables us to perceive the vertex’s ability to handle more flow. On the contrary, a negative value can cause flow blockage.

After adjusting the vertices’ capacities, we must adjust the edges’ capacities. For this, we calculate the inflow rate based on the effect of the arrival rate on the edges’ distribution probability (line 22). This is because the arrival rate affects not only the wards’ behaviours but also the frequency at which patients arrive at the edge. Then, we measure the effect of the inflow rate on the edges’ capacities (line 23). Hence, with a high arrival rate and high distribution probability, the edge may encounter bottlenecks if the proceeding vertex has high service time and low vertex capacity and number of servers. Finally, we sum up the adjusted capacities for each pair of vertex and incoming edge and store the value in the *StaRG* static residual graph (line 24).

After the capacity adjustment, the algorithm uses a depth-first search function to search for the available path for the flow to go through based on edge availability, direction, and positive capacity. This is because negative capacities are a sign of overflow, and the flow is blocked from passing through (lines 25 to 28). In a system like a hospital, issues can originate from any location, irrespective of the vertex significance, and patients are required to visit the necessary wards based on their health issues. Therefore, we decided to use depth-first search instead of breadth-first search and bottlenecks can appear in less priority wards, which may not be detectable using breadth-first search due to its nature of exploring shorter paths or higher priority vertices. The breadth-first search may overshadow the less priority wards, especially when there are high priority wards within the path. Using depth-first search, on the other hand, allows us to explore deeper into the path and identify less priority wards. Hence, it provides a more thorough analysis of potential flow blockages across the entire network. The function returns the available path for the algorithm to find the minimum residual capacity (line 29). To measure the flow, the algorithm uses the default measurement system (lines 30 to 31), with the difference that we eliminate the backward flow measurement. This is because, in hospitals, patients do not take alternative care pathways. Each patient follows a care pathway based on their health issue, and there is no alternative for their recovery process. The rest of the algorithm identifies the highest and lowest residual capacities and stores them in *DynRG* to observe the bottlenecks’ persistency, severity, and overflow, and the current residual capacities are stored in *DynV* to observe the dynamic nonlinear behaviours over time for each ward. The algorithm is based on a single-commodity flow where there is only one vertex to begin the flow and one vertex to end the flow.

### Verification and validation

To evaluate the algorithm, we use numerical data from Akademiska University Hospital previously analysed^38^ to model the hospital. We store the data in an Excel file and access them through code. The first adjacency list contains the wards’ names and the corresponding number of beds and staff. Due to the unavailability of staff numbers, we set the number of servers equal to the number of beds. The second adjacency list contains the edges with corresponding capacities and distribution probabilities. The capacities for all the edges are set to 5 with the presumption that a maximum of 5 patients are allowed to wait for bed availability in an upcoming ward. In the third list, we have stored the arrival rates per hour for 24 hours. Finally, we have a list that contains multiple lengths of stays for each ward, which is used to indicate proper distribution functions.

**Fig. 1:**
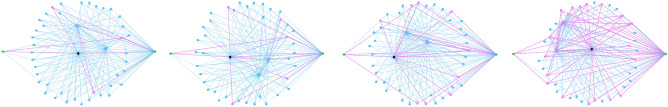
Patients’ flow evolution from left to right: 1 day, 1 week, 1 month and 1 year.

To validate, we gathered all the paths from the depth-first search function throughout the iteration to observe the paths the flow can reach, illustrated in Figure 1. The source vertex (left) and sink vertex (right) are coloured green, and the *Emergency Department* is coloured black. If the edges and vertices are coloured blue indicates there is a blockage for the flow to pass; otherwise, they are coloured purple. This can be due to significantly low distribution probability (e.g. $\approx 0.00003$ per hour), which prevents the flow from taking certain paths, or it can be due to significantly high service time (e.g. $\approx 1300$ hours) or low wards’ capacities (e.g. $\approx 2$ beds) which can cause negative adjusted capacity that indicates the corresponding wards are unable to handle more flow. Considering that some wards in a hospital only receive patients once a week, month, or year, we ran the algorithm for longer time intervals to observe how the flow evolves through the hospital over a year.

**Fig. 2:**
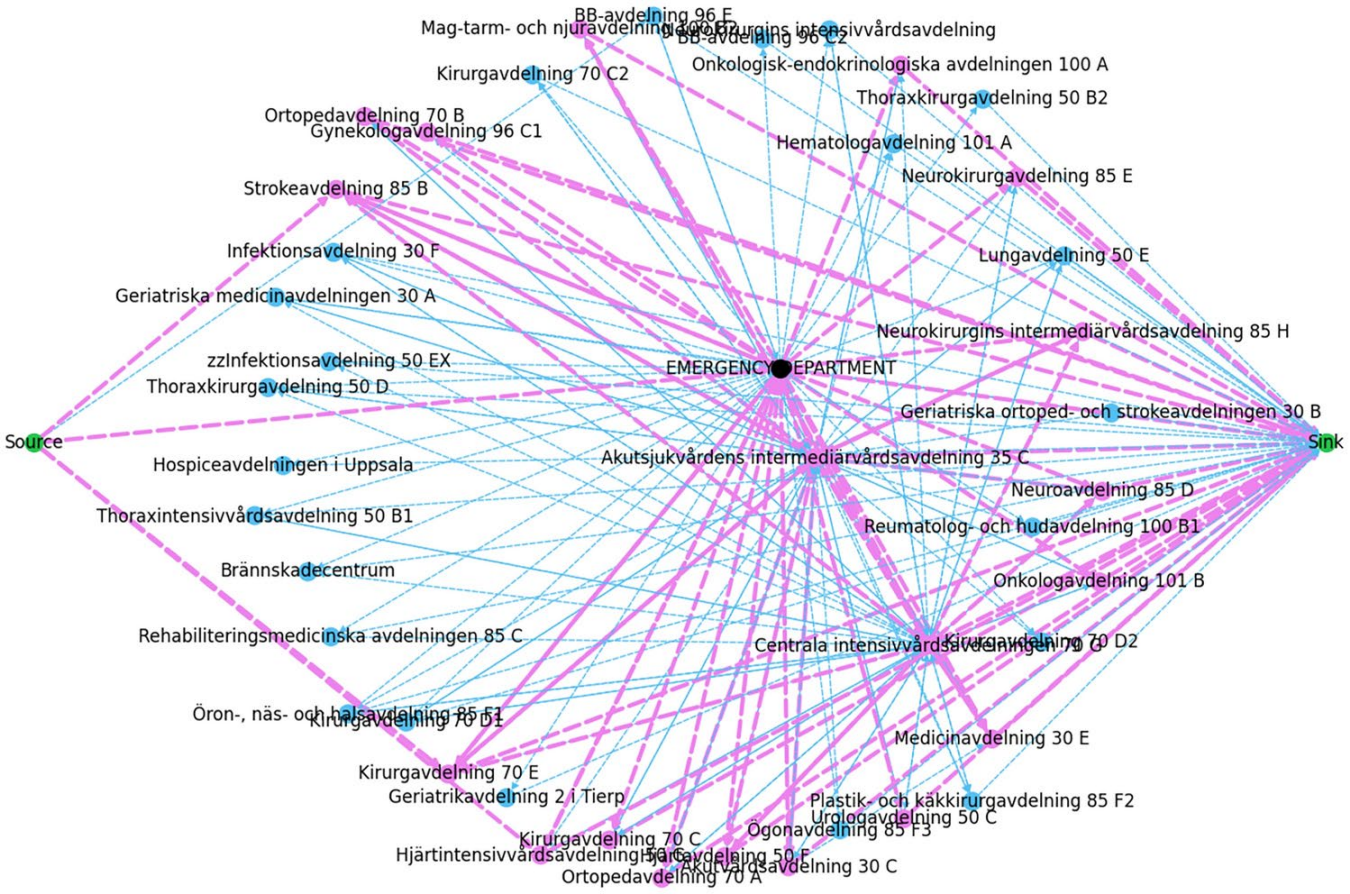
Patients’ flow for 1 year based on the hospital distribution probabilities.

**Fig. 3:**
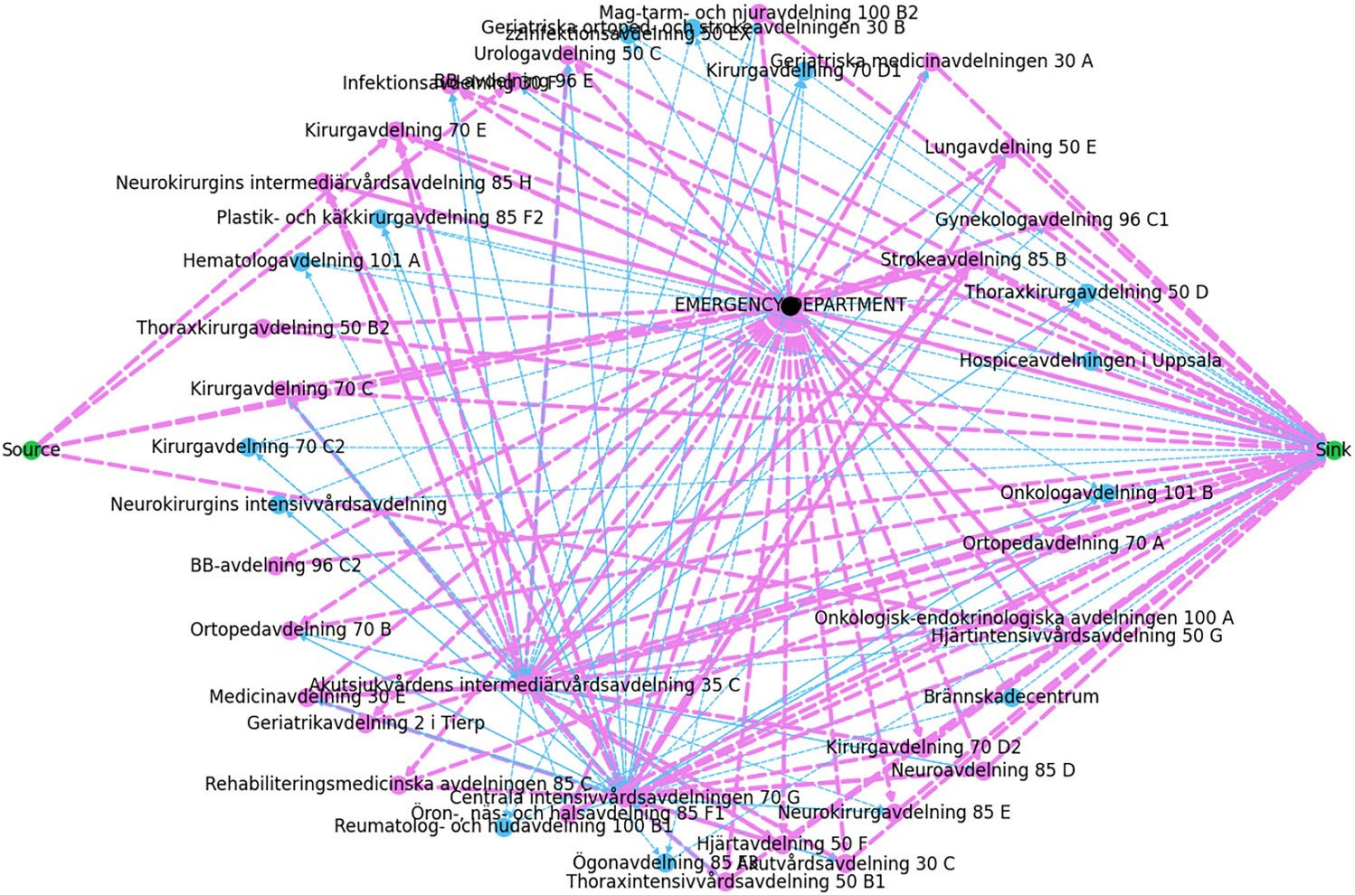
Patients’ flow over 1 year based on equal distribution probabilities of all the outgoing edges of a ward.

Despite running the algorithm for one year, Figure 2 indicates that 23 (may vary between [21,23]) out of 45 wards were still blocked. To uncover the reasoning behind the blockage, we performed a comparison analysis by substituting the distribution probabilities from hospital data with equally distributing the flow among all the outgoing edges per vertex. This is to ensure that the blockages are due to significantly low distribution probabilities and not the vertices’ capacities and service times. Therefore, we divided 1 with the number of outgoing edges per ward: $\frac{1}{|\text {Out}(u)|}$. To compare the results, the edges in Figure 3 indicate that only 13 out of 45 vertices are blocked, which approximately all are also blocked in both Figures 2 and 3. These vertices have significantly low capacities. This implies that the flow is not only blocked by vertices’ capacities but also by distribution probability. The remaining vertices which are still blocked have significantly low capacities in the range of [2,5], where few of them can have significantly high service times, causing negative adjusted capacities, which are unable to handle more flow.

**Fig. 4:**
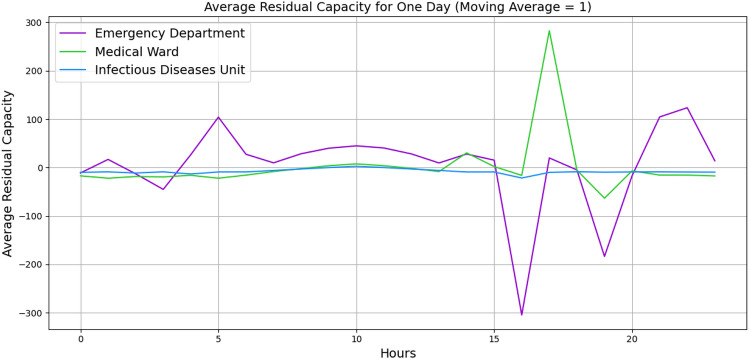
Average patients’ flow for 1 day duration using Akademiska Hospital distribution functions.

**Fig. 5:**
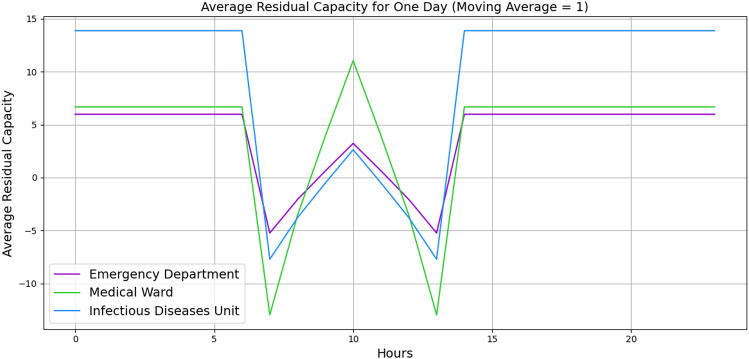
Average patients’ flow for 1 day duration using constant service times 5.01.

For further validation, we manually created a small-sized hospital with 20 vertices with the number of beds and staff and 62 edges with buffer size and distribution probabilities. A list of minimum required wards is listed, such as *Infectious diseases Unit*, *Emergency Department*, *Medical Ward*, *Cardiac*, *Respiratory* and etc. with a random number of beds and staff. The edges are given a buffer size of 5, and the distribution probabilities are given based on the importance of the ward. Figures 4 and 5 illustrate the behaviour of three wards. The x-axis indicates the hours, and the y-axis indicates the average residual capacity. The negative values indicate overflow, the positive values indicate that more flow still can go through, and zero means the vertices are at capacity but not yet overflowed. The aim is to represent the overflow of the wards when there is a high fluctuation of the patients’ arrival rate. Hence, we defined the following arrival rates: 1, 1, 1, 1, 1, 1, 1, 10, 20, 30, 40, 30, 20, 10, 1, 1, 1, 1, 1, 1, 1, 1, 1, 1. To define the service time, Figure 4 illustrates the wards’ behaviours based on Akademiska Hospital’s distribution functions, whereas Figure 5 illustrates the wards’ behaviours, given a constant service time of 5.01 (hours) across all wards. While both figures illustrate the change in their behaviour from time 7, the behaviour variation also depends on the distribution probability and the service time per patient. Additionally, the peak in Figure 5 at time 10 may indicate that patients leave the wards and other patients who were waiting enter the wards, while in Figure 4, the aggregation of patients causes overflow at time 15. The outlier values over +1000 and below -1000 are eliminated to observe the proper behaviour.

## Results

### Experiments

Table 1 contains the list of experiments using the Akademiska Hospital data. The first, second, and third experiments simulate the patients’ flow for a 1 day duration to observe the bottlenecks’ persistency, severity, and overflows of the hospital. Persistency allows us to observe the care pathways and wards’ behaviour stability and fluctuation based on the divergence of the minimum and maximum residual capacity; Severity allows us to observe the care pathways and wards that reach full capacity based on the subtraction of the maximum residual capacity from 0; and overflow is measured based on the subtraction of the minimum residual capacity from 0. These experiments allow us to analyse the bottlenecks and identify the root causes of the overflows. The fourth and fifth experiments simulate the patients’ flow for 1 day and 1 month duration to observe the dynamic nonlinear behaviour of the *Emergency Department*, *Medicinavdelning 30 E* and *Infektionsavdelning 30 F* wards using an equal or different number of servers and beds. The *Emergency Department* is known for being a priority ward, *Medicinavdelning 30 E* has fluctuating inflow and outflow, and *Infektionsavdelning 30 F* is vital in case of infectious diseases. These experiments will allow us to perceive the wards’ dynamic nonlinear behaviours for longer time intervals.

**Table 1:** Experiments.

Simulation Duration	Purpose	Outcome
1 day	Bottlenecks persistency	Figure 6
1 day	Bottlenecks severity	Figure 7
1 day	Overflows and root causes	Figure 8
1 day and 1 month	Wards behaviour with an equal number of servers and beds	Figures 9 and 10
1 day	Wards behaviour with a number of servers half the number of beds	Figure 11
1 day	Wards behaviour with a number of beds half the number of servers	Figure 12

### Bottlenecks’ persistency and severity

**Fig. 6:**
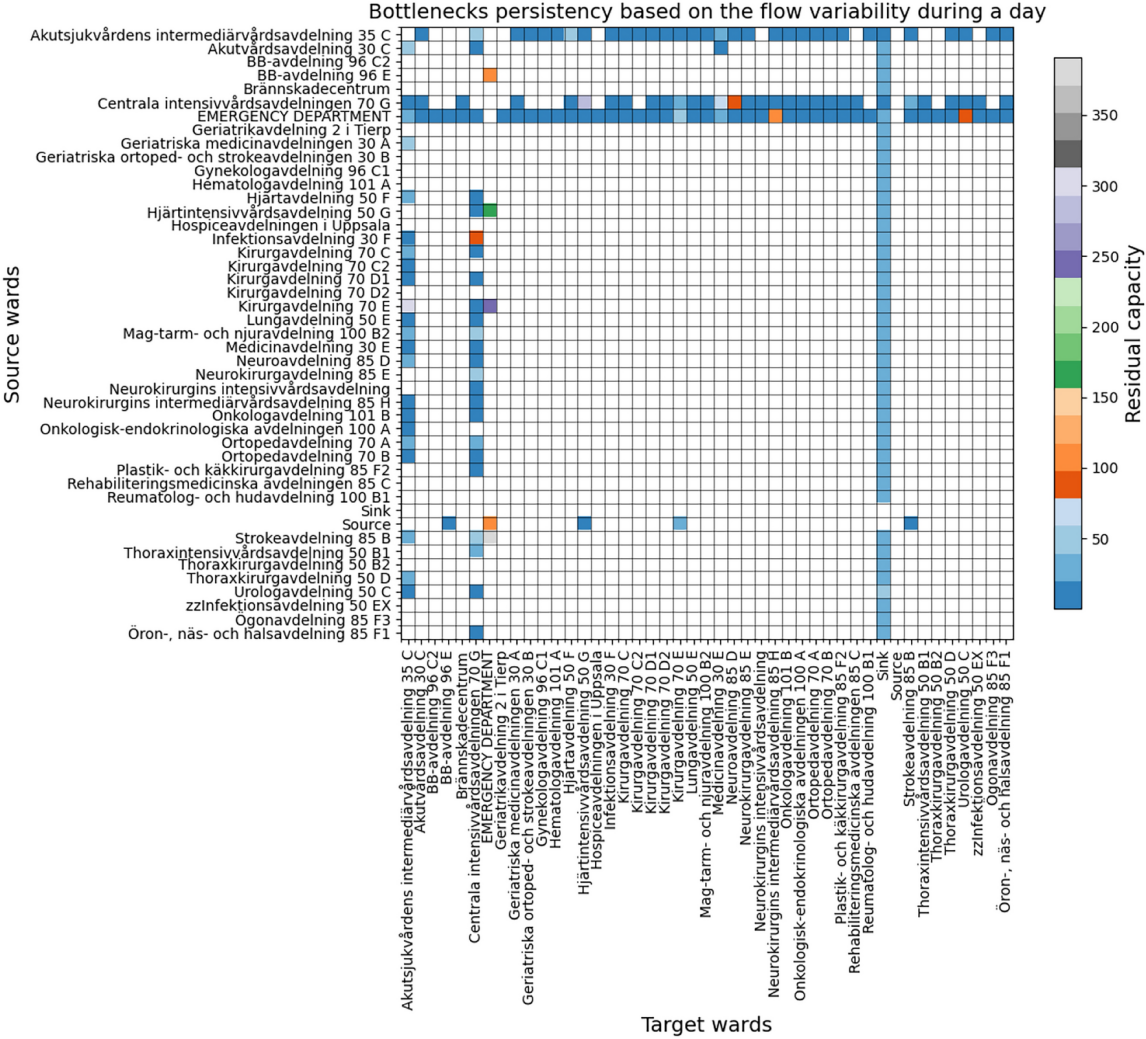
Bottlenecks’ persistency based on highest and lowest residual capacity divergence.

**Fig. 7:**
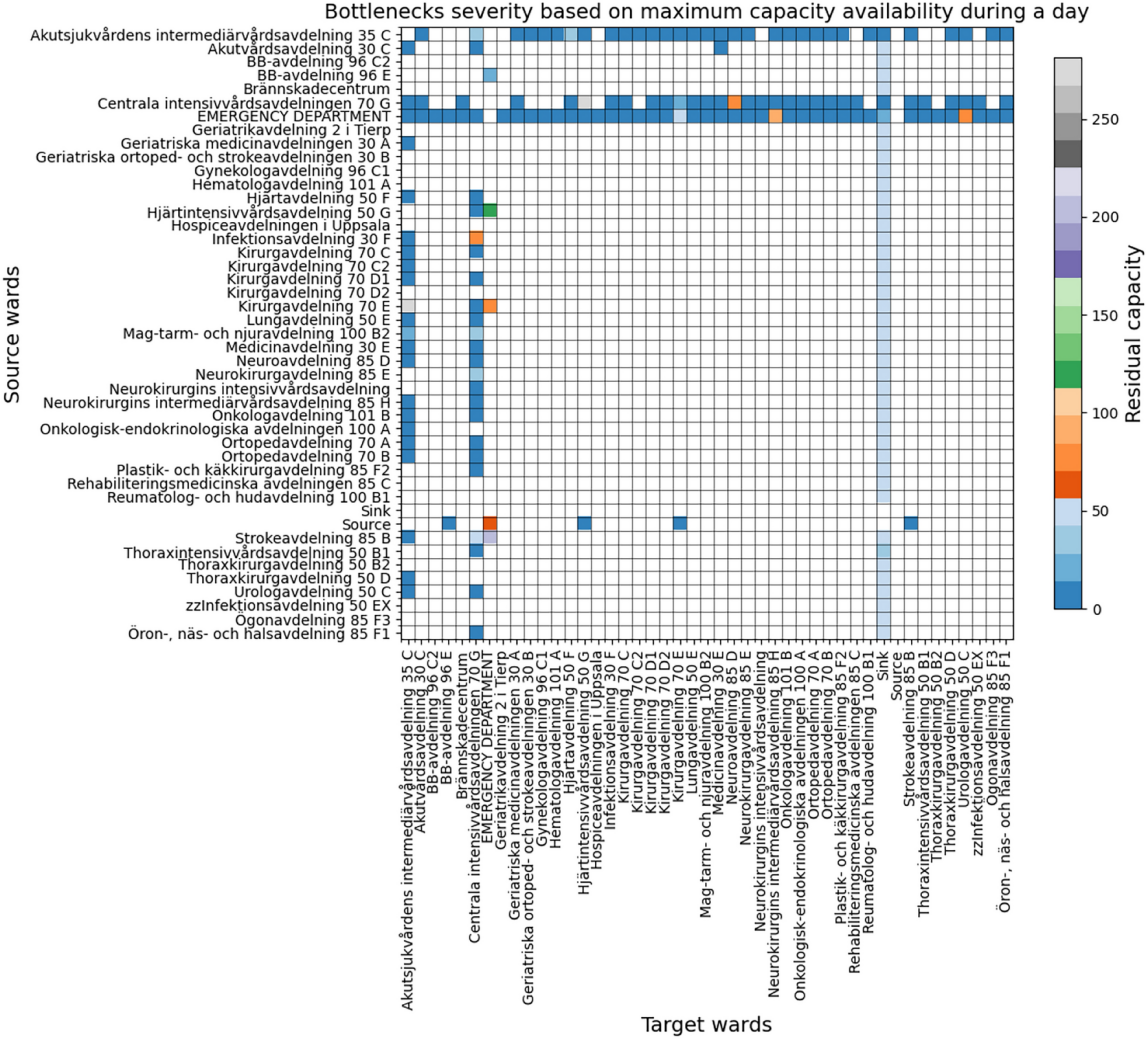
Bottlenecks’ severity based on the maximum residual capacity before reaching the full capacity.

Figures 6 and 7 illustrate the persistency and severity of the bottlenecks. The y-axis and the x-axis indicate the source and target vertices of the directed edges, where the empty cells indicate that there is no edge, and the residual capacity indicates the availability for more flow to pass through. The divergence indicates high fluctuations and instability in flow behaviour.

Figure 6 indicates that the majority of the edges have persistent flow within the range of [0,80]. However, there are fluctuations in the incoming flows to the *Emergency Department* than the outgoing flows. On the contrary, the incoming flows to the *Centrala Intensivvårdsavdelningen 70 G* have comparingly higher fluctuations than the outgoing flows.

**Fig. 8:**
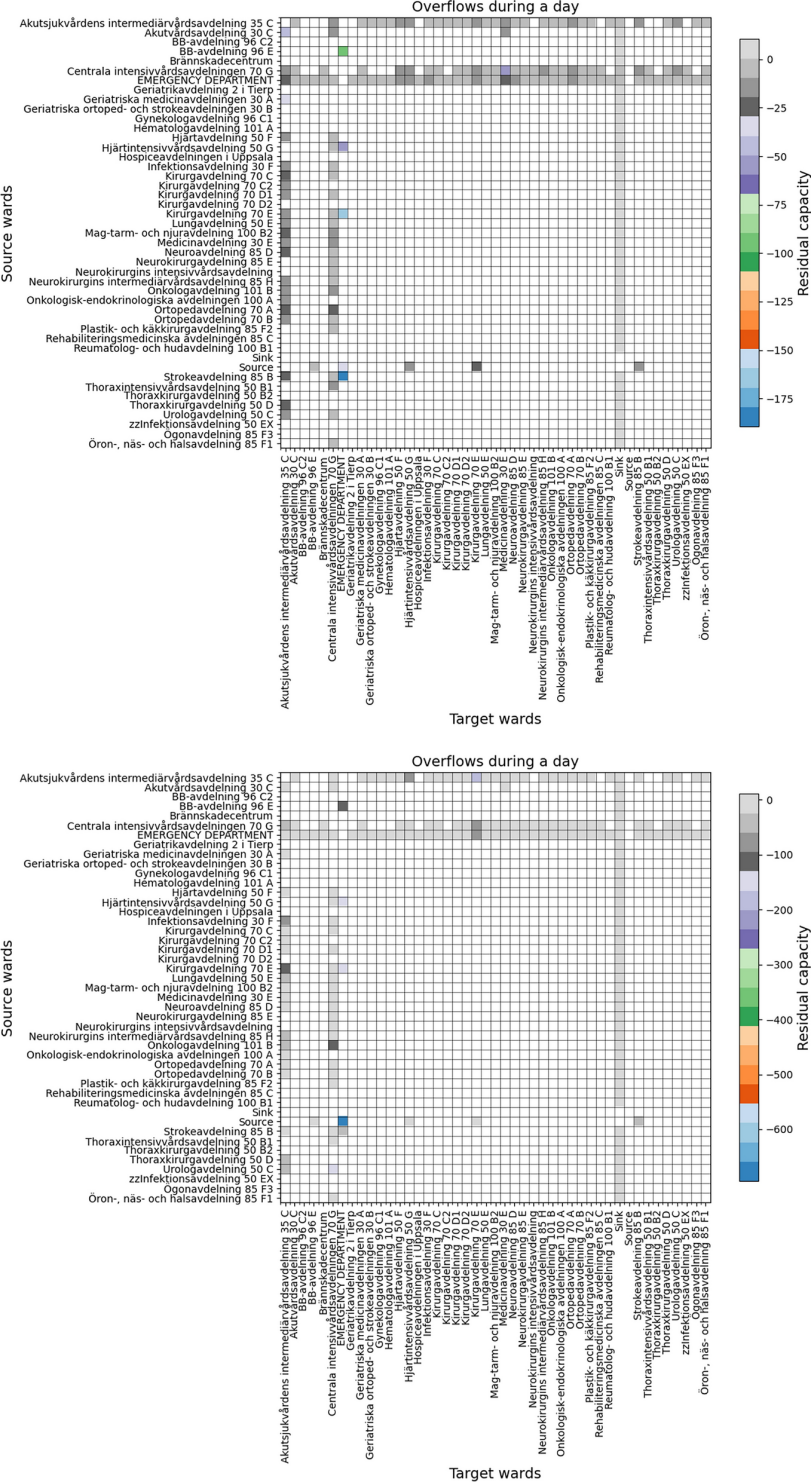
Overflows for 1 day in two different experiments.

Figure 7 indicates that the majority of the edges have severe bottlenecks. However, the incoming flows to the *Emergency Department* have less severe bottlenecks than the outgoing flows. This may imply that the *Emergency Department* performs well as the patients’ inflow rate is lower than the outflow rate; however, compared to the results in Figure 8, we can observe that the inflow rate can cause bottlenecks as well. This can be affected by the arrival rate rather than the service time within the *Emergency Department*, as the outflow behaviour is stable.

### Overflows and root causes

Figure 8 illustrates the hospital overflows. The figures indicate that the majority of the edges are reaching full capacity or are at full capacity. This is because each bed in each ward has one doctor to serve the patient, which reduces the workload. However, significant overflow exists toward both *Centrala Intensivvårdsavdelningen 70 G* and *Emergency Department* but less overflow toward other wards. There is also a significant overflow toward *Emergency Department* from the source vertex than other wards.

To identify the root causes of the bottlenecks, we trace the (e.g. second or third) predecessors or successors of the wards. Bottlenecks emerging from predecessors can be due to their high outflow rates pushing the flow into the current ward, and the bottlenecks emerging from successors can be due to their low outflow rates preventing flow blockage. For example, in Figure 8 (left), we can observe a significant flow from *Strokeavdelning 85 B* towards *Emergency Department*; by tracing the second predecessor, we can identify that a significant inflow from the source to *Strokeavdelning 85 B*. Similarly, there is a significant outflow from *Emergency Department* towards *Medicine Avdelning 30 E*, and a significant outflow from the *Centrala Intensivvårdsavdelningen 70 G* towards *Medicine Avdelning 30 E*. By tracing to other successors, we notice a low outflow rate from *Medicine Avdelning 30 E*, indicating that *Medicine Avdelning 30 E* receives more patients than it is able to handle.

### Wards’ dynamic nonlinear behaviours

**Fig. 9:**
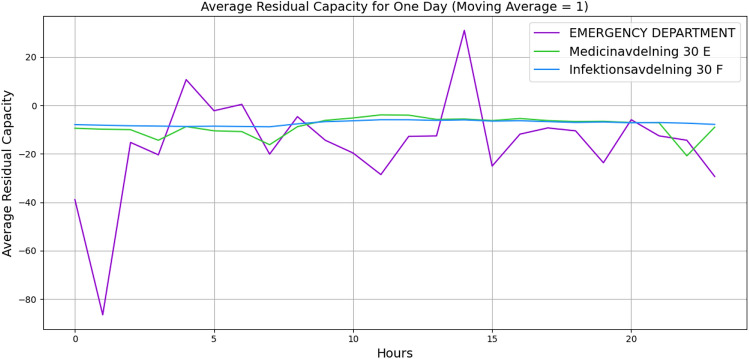
Average patients’ flow for 1 day duration.

**Fig. 10:**
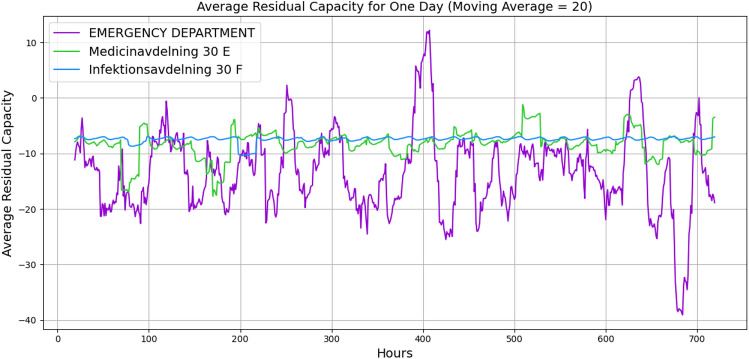
Average patients’ flow for 1 month duration.

**Fig. 11:**
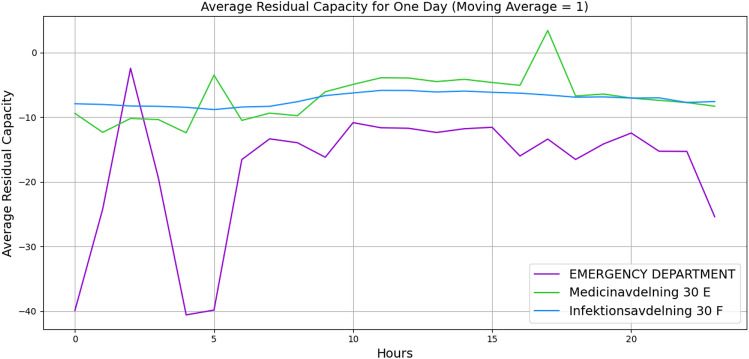
Average patients’ flow for 1 day duration when servers are half the number of beds.

**Fig. 12:**
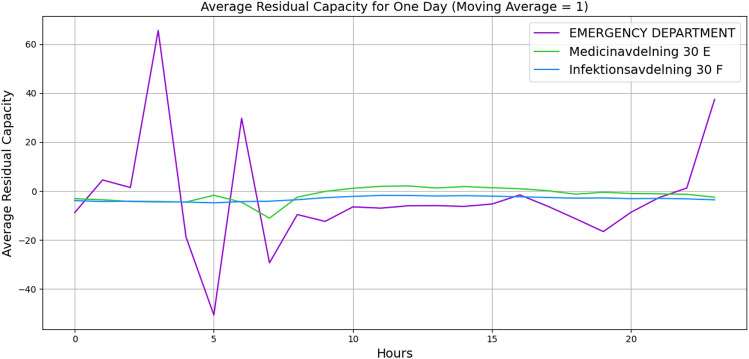
Average patients’ flow for 1 day duration when beds are half the number of servers.

Figures 9 and 10 illustrate the wards’ dynamic nonlinear behaviours for 1 day and 1 month, respectively. In both figures, the number of beds and servers are equal. The figures indicate that *Emergency Department* has significant perturbation, *Medicinavdelning 30 E* has moderate perturbation, and *Infektionsavdelning 30 F* has steady behaviour. Furthermore, the *Medicinavdelning 30 E* and the *Infektionsavdelning 30 F* have steadier overflow and persistent behaviour. As the flow continues for a 1-month duration, the *Emergency Department* continues to have a significant overflow. However, the *Medicinavdelning 30 E* shows moderate overflow and perturbation, and the *Infektionsavdelning 30 F* shows slight overflow and persistent behaviour. Additionally, Figures 11 and 12 illustrate the wards’ behaviour for 1 day where, in one scenario, the number of servers is half of the number of beds, and in the second scenario, the number of beds is half the number of servers. The figures illustrate that in all three wards, with more beds, there is significant overflow, and with more servers, there is compareingly less overflow and even capacity for more patients to flow through. This demonstrates that the algorithm is capable of properly illustrating the relationship between the number of servers and beds.

## Discussion and conclusion

In this paper, we proposed a dynamic nonlinear flow algorithm to model the patients’ flow through a hospital network where the vertices represent the wards, and the edges represent the care pathways and flow direction. The algorithm iterates over the patients’ arrival rate to measure the flow using the vertices’ capacities (beds), servers (staff), service time variability, edges’ capacities, and distribution probability to identify the bottlenecks’ persistency and severity, overflows and their root causes, and the wards dynamic nonlinear behaviour.

Our modified algorithm considers the arrival rate and service time variation and keeps the vertices’ capacities, vertices servers, edge capacity, and edge distribution probability fixed. The reason is that patients’ arrival can vary based on the time of the day, and the service time can vary based on their health issues and ward. However, in a hospital system, the number of staff can vary by attending to priority wards or patients over less prioritised wards and patients. This requires enhancing the algorithm by considering the servers’ variability, e.g., using a proper distribution function for server variability of wards.

The algorithm aims to provide a foundation to ensure that the model remains computationally feasible by prioritising the critical variables and conditions to illustrate the algorithm’s application for healthcare. The network’s edges are the hospital’s care pathways to indicate how the patients flow. Sometimes, patients have to repeat some procedures by going back to the previous wards^39^. In such cases, the network can include the edges with their corresponding distribution probabilities to indicate the proportion of the patients who should return to repeat procedures, depending on how detailed the model should be.

The algorithm is based on single-commodity, where the flow originates from a single source vertex and terminates in a single target vertex. This may vary if patients enter and leave from different locations based on their health issues with different arrival rates. To enhance the algorithm, multi-commodity flows could be taken into consideration. Finally, in hospitals, patients visit wards based on their health issues^4^. Here, specific types of patients are an abstraction that we include in the distribution probability. The distribution probability indicates the frequency of patients visiting certain wards over others, which is based on the patient’s health issue. Hence, adding specific health issues would be beneficial when it requires testing scenarios of blocking care pathways and wards or shifting patient flow to a different ward and seeing how it affects the hospital’s performance.

Our algorithm uses depth-first search instead of breadth-first search for path searching. This is because in a hospital, bottlenecks can originate in any ward irrespective of their priority, and the aim is to find available paths instead of the shortest paths. Using depth-first search prevents overshadowing the less priority wards by high priority wards, provides a complete list of paths, and is time-efficient. To enhance, the path searching algorithm can be tailored to patients’ disease types.

The algorithm offers a quick holistic view of a hospital’s performance to help decision-makers optimise and manage resources. This can be achieved by changing the number of beds in the wards with significant bottlenecks and comparing the results. Moreover, this can be implemented by considering the vertices’ capacities variability over time to reduce the bottlenecks. Additionally, the algorithm provides knowledge regarding the hospital’s overall performance for collaborative decision-making before further improvement or implementation of advanced models. Managing the resources in a priority ward may leave the issue intact in other wards. The algorithm can help to trace the path to where the bottleneck originates. For instance, suppose there are bottlenecks in the *Emergency Department*. Such bottlenecks can occur if there is a high outflow rate from one of the predecessor wards or a slow outflow rate in one of the successor wards. The predecessor ward may cause this if the service time is short, and the successor ward may cause this if the service time is long. Hence, by tracing the care pathways, we can identify the second and third predecessors or successors where the problem originates. Hence, the algorithm may help to shift decision-makers’ focus from a more priority ward to a less priority ward before financial investment in resource optimisation.

An advantage of this algorithm over other modelling approaches is that given the source(s) and target(s) vertices and edges direction, the flow initiates, terminates, augments the path, and measures the flow without the need to define queue disciplines. Furthermore, the algorithm is ready and quick to use before considering the use of simulation methods such as discrete-event simulation or agent-based network simulation, which can be time-consuming, computationally expensive, and more complex to implement. Finally, it can be adapted to directed networks that represent a hospital on any scale, such as clustering the wards into subsystems or extending the network with more wards to observe improvement in the bottlenecks.

Network algorithms quantify the vertices and edges in a graph, which can be challenging to interpret what the results indicate and may cause invalid conclusions. By default, the flow algorithm provides a discrete value of the remaining capacity (e.g., the number of available places in a road network or the number of available beds in a ward). However, here, we consider the effect of the intensity ratio on the capacity to observe the bottlenecks and overflow, which indicates the severity rate of the bottleneck and not the availability of the capacity. Hence, a negative residual capacity indicates the intensity of the overflow, and a positive residual capacity indicates the intensity of the bottleneck based on the space left for more flow.

To model a system using dynamic flow algorithms, we need to consider the required parameters and their effects. Applying such algorithms without the consideration of the limitations may produce invalid results. A generalised flow algorithm to optimise transportation or telecommunication cannot model the patients’ flow. For instance, to model public transportation using a dynamic flow algorithm, we can easily use the inflow and outflow rates based on the fixed schedule. However, in a hospital, due to the uncertainty of patients’ service time and arrival rates, we need to consider the effect of variability. Additionally, in a transportation network, edges’ capacities are crucial in handling traffic congestion, whereas, in a hospital, vertices’ capacities and servers are crucial to handle the bottlenecks. Therefore, our algorithm can be generalised to model other hospitals.

## Data Availability

The datasets used and/or analysed during the current study available from the corresponding author on reasonable request.

## Code availability

The code and the small hospital data are available here: Dynamic Nonlinear Flow Algorithm GitHub Repostory.

## References

[1] Bhattacharjee, P. & Ray, P. K. Patient flow modelling and performance analysis of healthcare delivery processes in hospitals: A review and reflections. *Computers & Industrial Engineering.* **78**, 299–312 (2014).

[2] Hall, R., Belson, D., Murali, P. & Dessouky, M. *Modeling patient flows through the healthcare system* (Reducing delay in healthcare delivery, Patient flow, 2006).

[3] Martínez-García, M. & Hernández-Lemus, E. Health systems as complex systems. *American Journal of Operations Research*. **3** (1A), 113–126 (2013).

[4] Gupta, D. & Denton, B. Appointment scheduling in health care: Challenges and opportunities. *IIE transactions*. **40** (9), 800–819 (2008).

[5] Ahmadi-Javid, A., Jalali, Z. & Klassen, K. J. Outpatient appointment systems in healthcare: A review of optimization studies. *European Journal of Operational Research*. **258** (1), 3–34 (2017).

[6] Berg, B. & Denton, B. T. Appointment planning and scheduling in outpatient procedure centers. In *Handbook of healthcare system scheduling*. (Springer, 2011).

[7] Williams, M. *Hospitals and clinical facilities, processes and design for patient flow*. (Reducing Delay in Healthcare Delivery, Patient Flow, 2006).

[8] Kolker, A. *Interdependency of hospital departments and hospital-wide patient flows*. (Reducing delay in healthcare delivery, Patient flow, 2013).

[9] Zacharias, C. & Pinedo, M. Managing customer arrivals in service systems with multiple identical servers. *Manufacturing & Service Operations Management*. **19** (4), (2017).

[10] Yadav, G. & Babu, S. Nexcade: perturbation analysis for complex networks. *PloS one*. **7** (8), e41827 (2012). 10.1371/journal.pone.0041827 PMC3411682 22870252

[11] Fone, D. et al. Systematic review of the use and value of computer simulation modelling in population health and health care delivery. *Journal of Public Health*. **25** (4), 325–335 (2003). 10.1093/pubmed/fdg075 14747592

[12] Williamson, D. P. *Network flow algorithms*. (Cambridge University Press, 2019).

[13] Buzna, L. & Carvalho, R. Controlling congestion on complex networks: fairness, efficiency and network structure. *Scientific reports*. **7** (1), (2017). 10.1038/s41598-017-09524-3 PMC5567293 28831116

[14] Goldberg, A. V., Tardos, É. & Tarjan, R. *Network flow algorithm*. (Cornell University Operations Research and Industrial Engineering, Tech. Rep., 1989).

[15] Skutella, M. An introduction to network flows over time. *Research Trends in Combinatorial Optimization: Bonn 2008* (2009).

[16] Green, L. Queueing analysis in healthcare. *Patient flow: reducing delay in healthcare delivery*. (2006).

[17] Jensen, K. Emergency department crowding: the nature of the problem and why it matters. *Patient flow: reducing delay in healthcare delivery* (2013).

[18] Wilkinson, W. L. An algorithm for universal maximal dynamic flows in a network. *Operations Research*. **19** (7), 1602–1612 (1971).

[19] Kosnik, L. *Breakthrough demand-capacity management strategies to improve hospital flow, safety, and satisfaction* (Reducing Delay in Healthcare Delivery, Patient Flow, 2006).

[20] Ford, L. R. & Fulkerson, D. R. *Flows in networks* (Princeton University Press, Tech. Rep., 1962).

[21] Ford Jr, L. R. & Fulkerson, D. R. Constructing maximal dynamic flows from static flows. *Operations research*. **6** (3), 419–433 (1958).

[22] Minieka, E. Maximal, lexicographic, and dynamic network flows. *Operations Research*. **21** (2), 517–527 (1973).

[23] Pyakurel, U., Nath, H. N., Dempe, S. & Dhamala, T. N. Efficient dynamic flow algorithms for evacuation planning problems with partial lane reversal. *Mathematics*. **7** (10), 993 (2019).

[24] Halpern, J. A generalized dynamic flows problem. *Networks*. **9** (2), 133–167 (1979).

[25] Kotnyek, B. An annotated overview of dynamic network flows. *Name of the Journal*. (2003).

[26] Minieka, E. Dynamic network flows with arc changes. *Networks*. **4** (3), 255–265 (1974).

[27] Bookbinder, J. H. & Sethi, S. P. The dynamic transportation problem: A survey. *Naval Research Logistics Quarterly*. **27** (1), 68–87 (1980).

[28] Como, G. On resilient control of dynamical flow networks. *Annual Reviews in Control*. **43**, 80–90 (2017).

[29] Nilsson, G., Como, G. & Lovisari, E. On resilience of multicommodity dynamical flow networks. In *53rd IEEE Conference on Decision and Control* (2014).

[30] Crielaard, L. et al. Using network analysis to identify leverage points based on causal loop diagrams leads to false inference. *Scientific reports*. **13** (1), 21046 (2023). 10.1038/s41598-023-46531-z PMC10687004 38030634

[31] Liu, Y., Liu, Y., Liu, F., Fan, J. & Tao, Z. An algorithm for discovering vital nodes in regional networks based on stable path analysis. *Scientific Reports*. **13** (1) (2023). 10.1038/s41598-023-39174-7 PMC10505182 37717092

[32] Aronson, J. E. A survey of dynamic network flows. *Annals of Operations Research*. **20**, 1–66 (1989).

[33] Fleischer, L. & Tardos, E. Efficient continuous-time dynamic network flow algorithms. *Operations Research Letters*. **23**, 71–80 (1998).

[34] Philpott, A. B. Continuous-time flows in networks. *Mathematics of Operations Research*. **15**, 640–661 (1990).

[35] Orda, A. & Rom, R. On continuous network flows. *Operations Research Letters*. **17**, 27–36 (1994).

[36] Fleischer, L. & Skutella, M. Minimum cost flows over time without intermediate storage. In *SODA*. (2003).

[37] Edmonds, J. & Karp, R. M. Theoretical improvements in algorithmic efficiency for network flow problems. *Journal of the ACM (JACM)*. (1972).

[38] Marzano, L. et al. Diagnosing an overcrowded emergency department from its electronic health records. *Scientific Reports*. **14**, 9955 (2024). 10.1038/s41598-024-60888-9 PMC11061188 38688997

[39] Gamarnik, D. & Shah, D. & Wei, Y (Convergence and correctness. Operations research, Belief propagation for min-cost network flow, 2012).

